# Supplementary material for: Allnighter pseudokinase-mediated feedback links proteostasis and sleep in Drosophila
Source: Nat Commun. 2023 May 22;14:2932. doi: 10.1038/s41467-023-38485-7 (PMC10203134; doi:10.1038/s41467-023-38485-7)

Supplementary Data for

Allnighter pseudokinase-mediated feedback links proteostasis and sleep in *Drosophila*

Shashank Shekhar[1,#], Andrew T. Moehlman[1,2], Brenden Park[3], Michael Ewnetu[1], Charles Tracy[1], Iris Titos[4], Krzysztof Pawłowski[3,5], Vincent S Tagliabracci[3,6], and Helmut Krämer[1,7,#]

Affiliations:

[1] Department of Neuroscience, UT Southwestern Medical Center, Dallas, TX; O'Donnell Brain Institute

[2] Current address: Surgical Neurology Branch, National Institute of Neurological Disorders and Stroke, National Institutes of Health, Bethesda, MD

[3] Department of Molecular Biology UT Southwestern Medical Center, Dallas, TX,

[4] Molecular Medicine Program, University of Utah, School of Medicine, Salt Lake City, UT

[5] Department of Biochemistry and Microbiology, Institute of Biology, Warsaw University of Life Sciences, Warsaw 02-776, Poland

[6] Howard Hughes Medical Institute

[7] Department of Cell Biology, UT Southwestern Medical Center, Dallas, TX.

# Correspondence to:

Shashank.Shekhar@UTSouthwestern.edu or

helmut.kramer@utsouthwestern.edu

Lead contact:

helmut.kramer@utsouthwestern.edu

Supplementary Figure 1

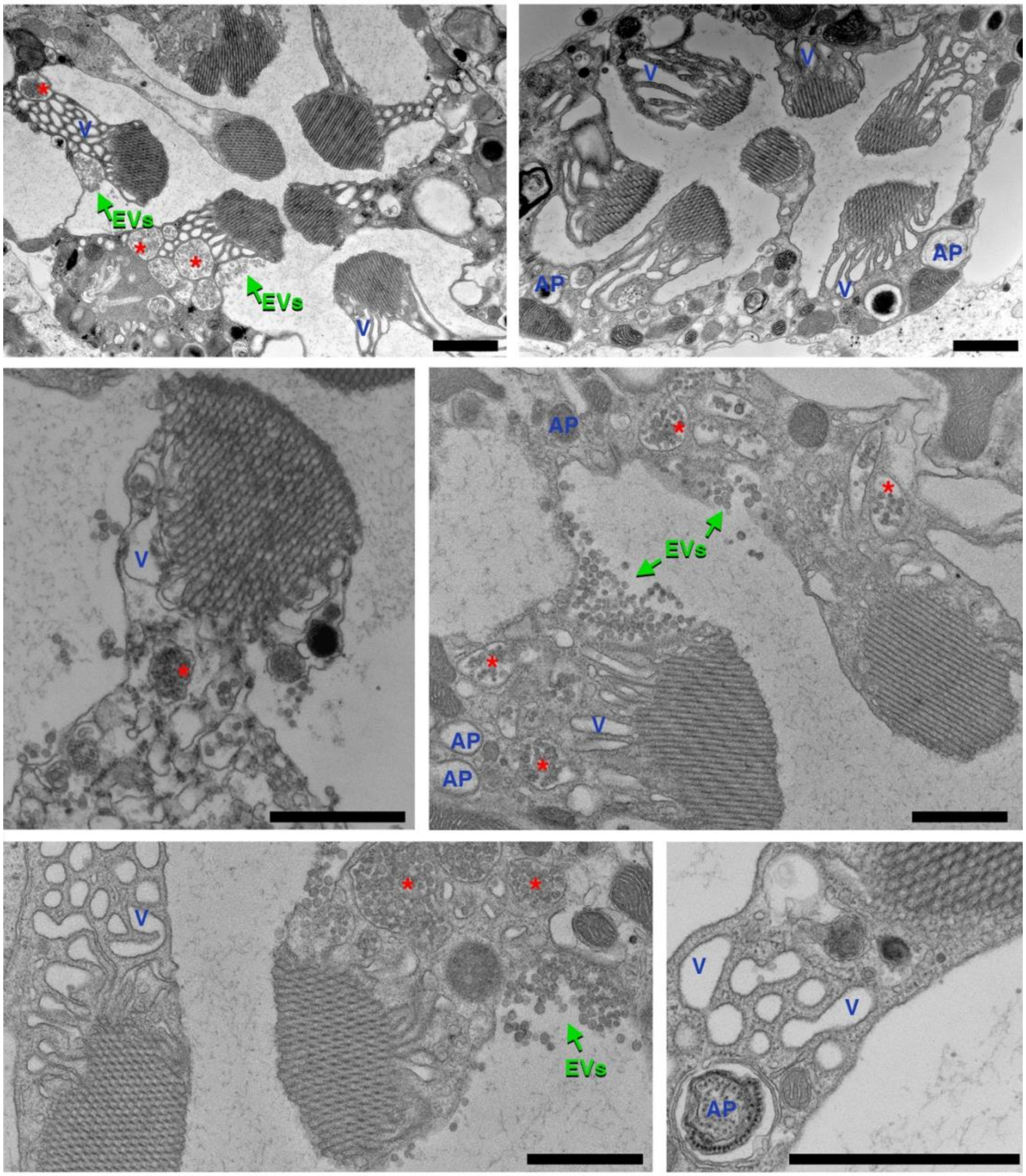

Supplementary Figure 1. Examples of *aln*[2] photoreceptor dysmorphology in LL.
A collage of electron micrographs depicts the variety of *aln*[2] photoreceptor dysmorphologies after 3 days of LL. Images show the aberrant presence of large vacuoles (V), autophagic vacuoles (AP), MVBs (*), and extracellular vesicles (EVs). Scale bars are 1 µm. TEM was repeated 3 times.

Supplementary Figure 2

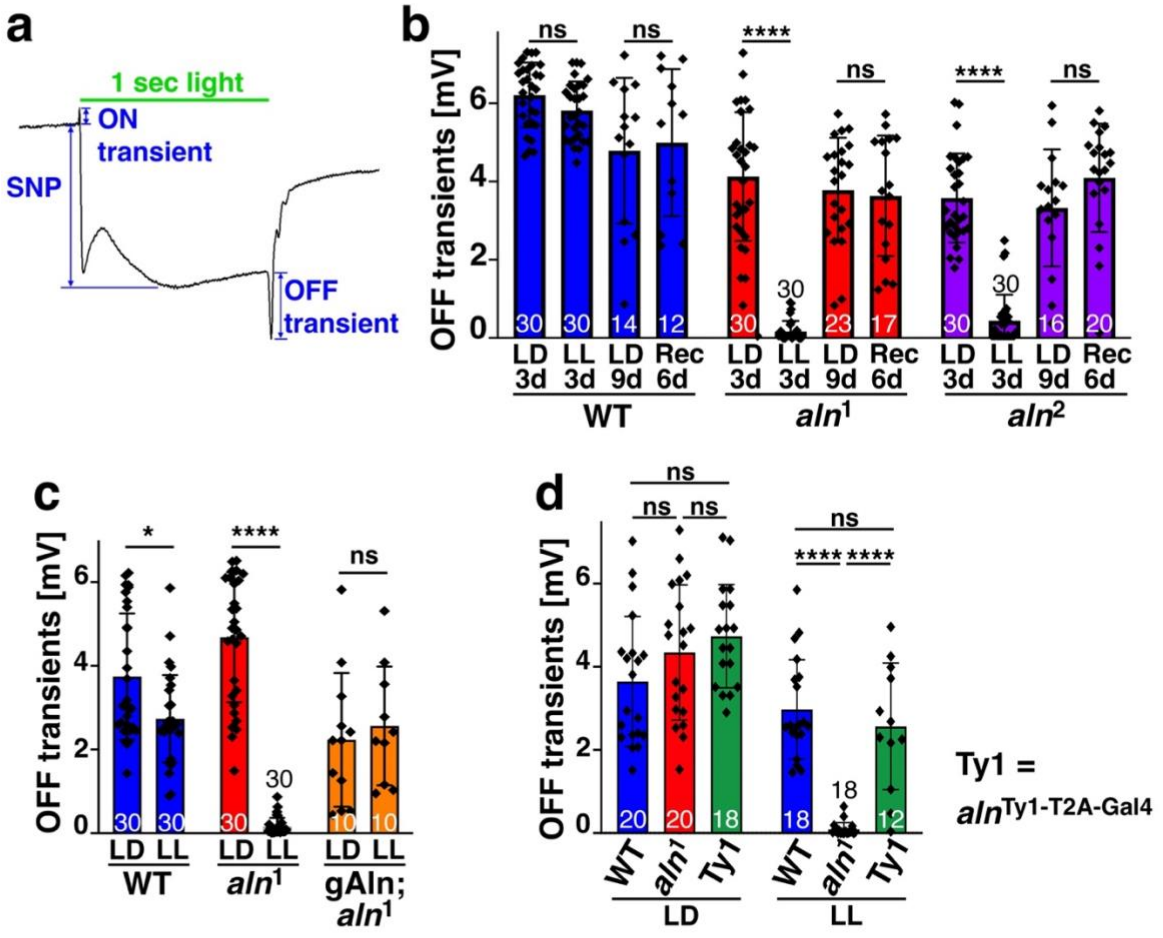

Supplementary Figure 2. ERG analysis of *aln* alleles.

(a) Diagram depicting typical wild-type ERG recording pointing to sustained negative potential (SNP), On transients, and OFF transient responses to a 1 sec light pulse.

(b) Quantification of OFF transients from ERG recordings of *aln*^{1} and *aln*^{2} mutants compared to wild-type controls show the same defect in maintaining OFF-transients after 3 days of LL and the same characteristic of their return upon recovery in 6 day of LD (Rec) compared to flies maintained for 9 days under LD only. P-values determined by two-way ANOVA followed by Bonferroni correction for multiple comparison test are for WT, LD-LL: 0.99, Rec-9dLD: 0.99; *aln*^{1}, LD-LL: 0.0001, Rec-9dLD: 0.99; *aln*^{2}, LD-LL: 0.0001, Rec-9dLD: 0.362. F (3, 270) = 71.05

(c) A genomic Aln transgene rescues the *aln*^{1} OFF transients under LL as shown in quantification of ERGs of wild type, *aln*^{1} and gAln*; aln*^{1} flies. P-values determined by two-way ANOVA followed by Bonferroni correction for multiple comparison test are for WT: 0.035; *aln*^{1}: <0.0001; *aln*$^{Ty1\text{-}T2A\text{-}Gal4}$: 0.99. Under LL: *aln*^{1} vs *aln*$^{Ty1\text{-}T2A\text{-}Gal4}$: <0.0001.

(d) Quantification of OFF transients from ERGs of wild-type, *aln*^{1} and *aln*$^{Ty1\text{-}T2A\text{-}Gal4}$ flies shows wild-type-like behavior of *aln*$^{Ty1\text{-}T2A\text{-}Gal4}$ flies with respect to loss of OFF transients under LL. Compared to WT, P-values determined by two-way ANOVA followed by Bonferroni correction for multiple comparison test are for LD, *aln*^{1}: 0.307; *aln*$^{Ty1\text{-}T2A\text{-}Gal4}$: 0.0907; for LL: *aln*^{1}: <0.0001; *aln*$^{Ty1\text{-}T2A\text{-}Gal4}$: 0.695.

(b-d) Significance threshold for p-Value are: ns, non-significant; *<0.05; **<0.01; ***<0.001; ****<0.0001. Genotypes are listed in Supplementary File 2.

Supplementary Figure 3

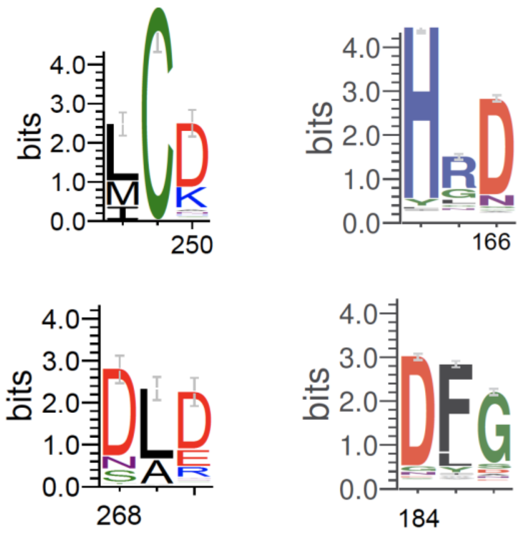

Supplementary Figure 3. Key kinase residues are not conserved in Aln.

Sequence logos are shown for insect homologs of Allnighter (left) and canonical kinases (right) Top row shows motifs corresponding to the HRD motif of canonical kinases, containing the catalytic D^{166} (substituted by K^{250} in Allnighter).
Bottom row shows motifs corresponding to the DFG motif of canonical kinases, containing the metal-binding D^{184} (substituted by S^{268} in Allnighter).
The logos were generated using the Weblogo algorithm [1].

Supplementary Figure 4

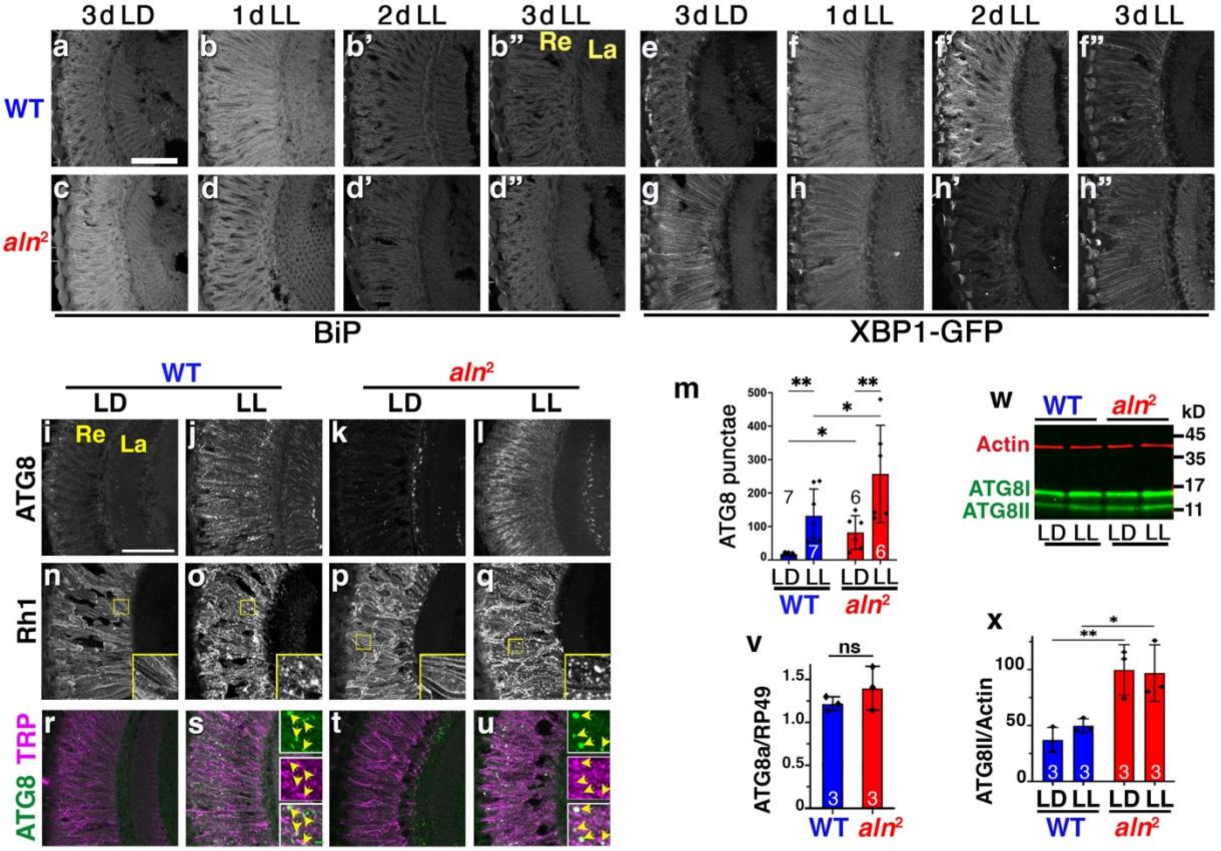

Supplementary Figure 4. Dysregulated stress responses in *aln* mutants.

(a-h) Time course of ER stress responses are visualized by staining for BiP or XBP1-GFP as shown in (a-h) Projection of confocal micrographs of cryosection of adult eyes of w^{1118} (a,b,e,f) or aln^2 (c,d,g,h) flies stained for BiP (a-d), or XBP1-GFP (e-h). Scale bar in a is 50 µm and same for a-h. Experiments depicted in a-h were repeated 3 times.

(i-u) LL-dependent induction of autophagy (i,l,n-u) is shown in projection of confocal micrographs of cryosection of adult eyes of w^{1118} (i,j,n,o,r,s) or aln^2 (k,l,p,q,t,u)) flies stained for ATG8 (i-l), Rh1 (n-q), or Atg8 and TRP (r-u). Flies were treated for 3 days at LD or LL as indicated. Scale bar in i is 50 µm and the same for i-u. Insets in n-q highlight the change in Rh1 distribution from rhabdomeres to vacuoles (See also Supplementary Figure 1). Insets in s,u highlight with arrowheads the colocalization of ATG8 and the rhabdomere protein TRP. Experiments depicted in i-u were repeated 3 times.

(m) Quantification of Atg8 punctae in the retinas of the indicated genotypes with bar graphs showing number of independent retinas and mean ±SD. P-values determined by two-way ANOVA followed by Tukey's correction for multiple comparison test are for WT, LD-LL: 0.0014; aln^2, LD-LL: 0.0014; LD, WT- aln^2: 0.041; LL, WT- aln^2: 0.041.

(v) Transcript levels for Atg8 normalized to ribosomal protein RP49 transcripts in w^{1118} or aln^2 flies with bar graphs showing number of independent experiments and mean ±SD. Two-tailed t-test p=

(w,x) Western blot of lysates from adult heads of w^{1118} or aln^2 flies. Flies were treated with LD or LL as indicated. Blots were stained for ATG8 and actin. (x) Quantification of lipidated Atg8II relative to actin. P-values determined by ANOVA followed by Sidak's correction for multiple comparison test are: LD, WT- aln^2: 0.0026; LL, WT- aln^2: 0.0176. Bar graphs show n and mean ±SD.

Significance threshold for P-Values shown in (m,v,x) are: ns, non-significant; *<0.05; **<0.01.

Supplementary Figure 5

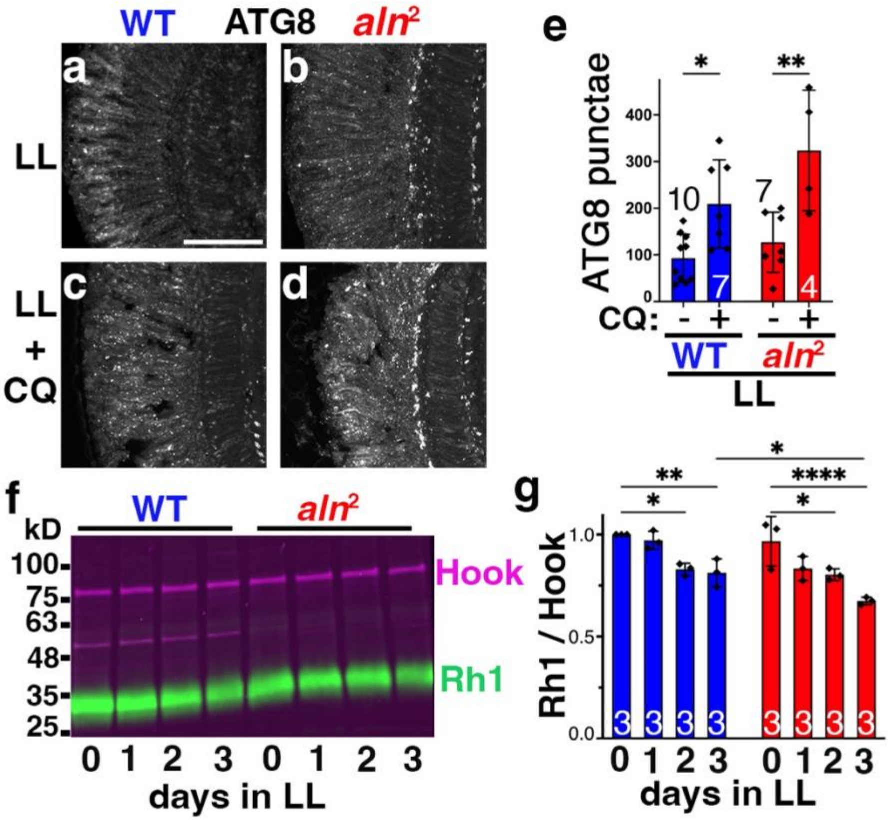

Supplementary Figure 5. Autophagy flux in *aln*^{2} retinas.

LL-induced Atg8 accumulation is not due to inhibition of autophagic flux as shown by increased staining upon inhibition of lysosomal degradation with chloroquine.

(a-d) Flies were LL-treated with or without 3 mg/ml chloroquine in their food. Diagrams show projections of confocal micrographs of cryosections of adult eyes of w^{1118} (a,c) or *aln*^{2} (b,d) flies stained for ATG8 (a-d). Scale bar in a: 50 µm and same for a-d.

(e) Bar graphs show quantification of Atg8 punctae in the retinas of WT and *aln*^{2} LL-treated flies with or without chloroquine from images as shown in A-D. Bar graphs show n and mean ±SD. P-values determined by two-way ANOVA followed by Tukey's correction for multiple comparison test are for WT: 0.0322; *aln*^{2}: 0.0032.

(f,g) Western blot of lysates from adult heads of w^{1118} or *aln*^{2} flies. Flies were treated for the indicated number of days under LL. Blots were stained for Rh1 and Hook for control. (g) Quantification of Rh1 relative to Hook. Bar graphs show n and mean ±SD. P-values determined by two-way ANOVA followed by Tukey's correction for multiple comparison test are for WT, 2d: 0.012, 3d: 0.0057; *aln*^{2} 2d: 0.0159, 3d: <0.000; 3d WT- *aln*^{2}: 0.0401.

Significance threshold for P-Values shown in (m,v,x) are: ns, non-significant; *<0.05; **<0.01.

Supplementary Figure 6

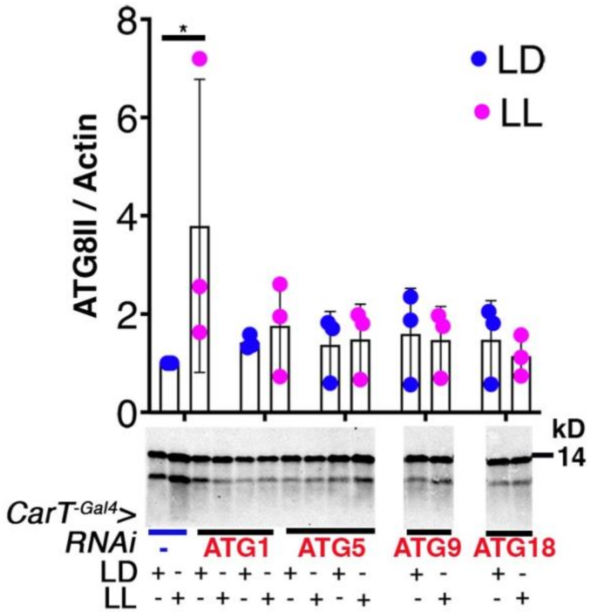

Supplementary Figure 6. LL-induced ATG8 activation depends on conventional autophagy.

Expression of RNAi-transgenes were used to knockdown expression of autophagy genes critical for several key steps in autophagy. Western blot and quantifications of lysates from adult heads of flies expressing the indicate UAS-RNAi-transgenes under control of the photoreceptor-specific $CarT^{HA\text{-}T2A\text{-}Gal4}$ driver and treated for three days under LD or LL. Blots were stained for Atg8 and Actin (not shown). Quantification of lipidated Atg8II relative to Actin (n= 3). Bar graphs show mean ±SD. P-values determined by two-way ANOVA followed by Sidak's correction for multiple comparison test are for no RNAi: 0.0340. Significance threshold for P-Values is: *<0.05.

Supplementary Figure 7

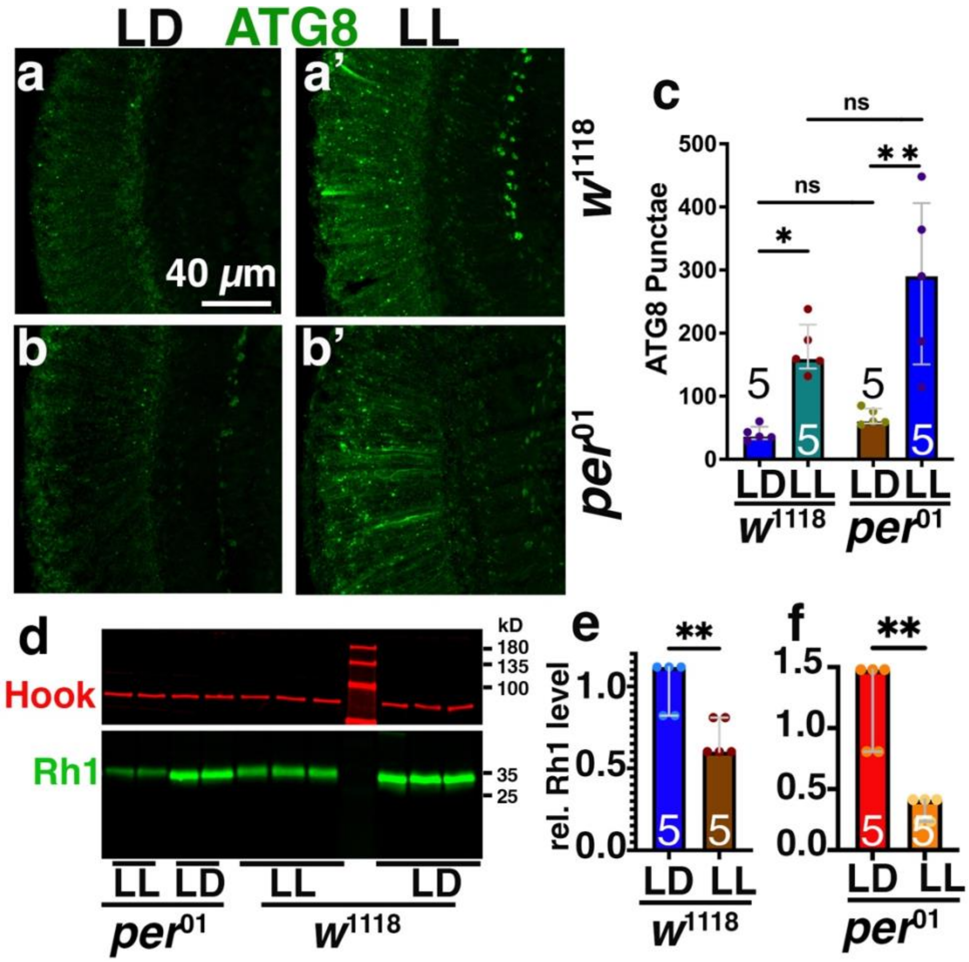

Supplementary Figure 7. LL-induced phenotypes of *per*^{01} mutants.

The circadian rhythm mutant *per*^{01} displays LL-induced phenotypes similar to wild-type flies.

(a,b) Head sections of wild-type (a) or *per*^{01} (b) LD or LL-treated flies were stained for Atg8. Scale bar (40 µm) in a is the same for a'-b'.

(c) Bar graphs show quantification of Atg8 punctae in the retinas of the indicated genotypes from images as shown in a,b. Bar graphs show number of fly heads and median with interquartile range. P-values determined by two-way ANOVA followed by Bonferroni correction for multiple comparison test are WT: 0.0497; *per*^{01}: 0.0012; LD: 0.99; LL: 0.182. F=12.15.

(d) Western blot of lysates from adult heads of *w*^{1118} or *per*^{01} flies. Flies were treated for the three days under LD or LL. Blots were stained for Rh1 and Hook for control.

(e,f) Quantification of Rh1 relative to Hook from *w*^{1118} (e) and *per*^{01} (f) as seen in (d). Bar graphs n independent samples from two experiments and median with interquartile range. P values determined by nonparametric Mann-Whitney test were (e) 0.0079 (f) 0.0079.

Significance threshold for P-Values shown in (c,e,f) are: ns, non-significant; *,<0.05; **,<0.01.

Supplementary Figure 8

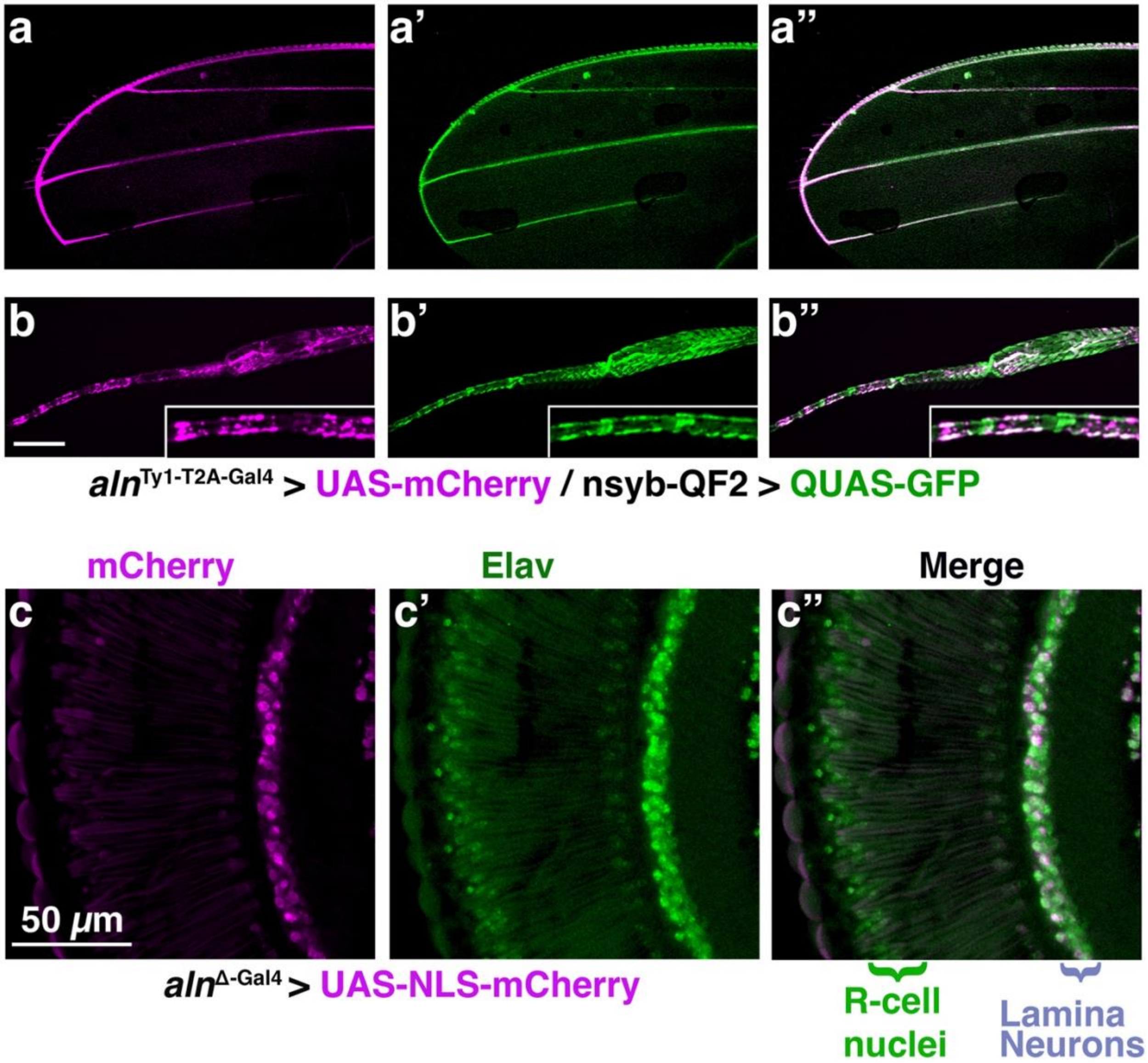

Supplementary Figure 8. Aln is expressed in peripheral sensory neurons and some lamina neurons.

Micrographs of a wing (a) and leg (b) from flies expressing Aln$^{Ty1\text{-}T2A\text{-}Gal4}$-driven UAS-mCherry and nSyb-QF2 driven QUAS-GFP. Colocalization of Aln expression with neuronal Synaptobrevin expressing cells indicates neuronal expression of Aln in peripheral sensory neurons. Scale bar in b is 200 µm and same for a-b". (c) Head sections of flies expressing uas-NLS-mCherry under *aln*$^{\Delta\text{-Gal4}}$ control stained for mCherry and Elav show aln-expressing neurons in the lamina, but not in the retina. Scale bar in c is 50 µm and same for c-c". Experiments were repeated twice.

Supplementary Figure 9

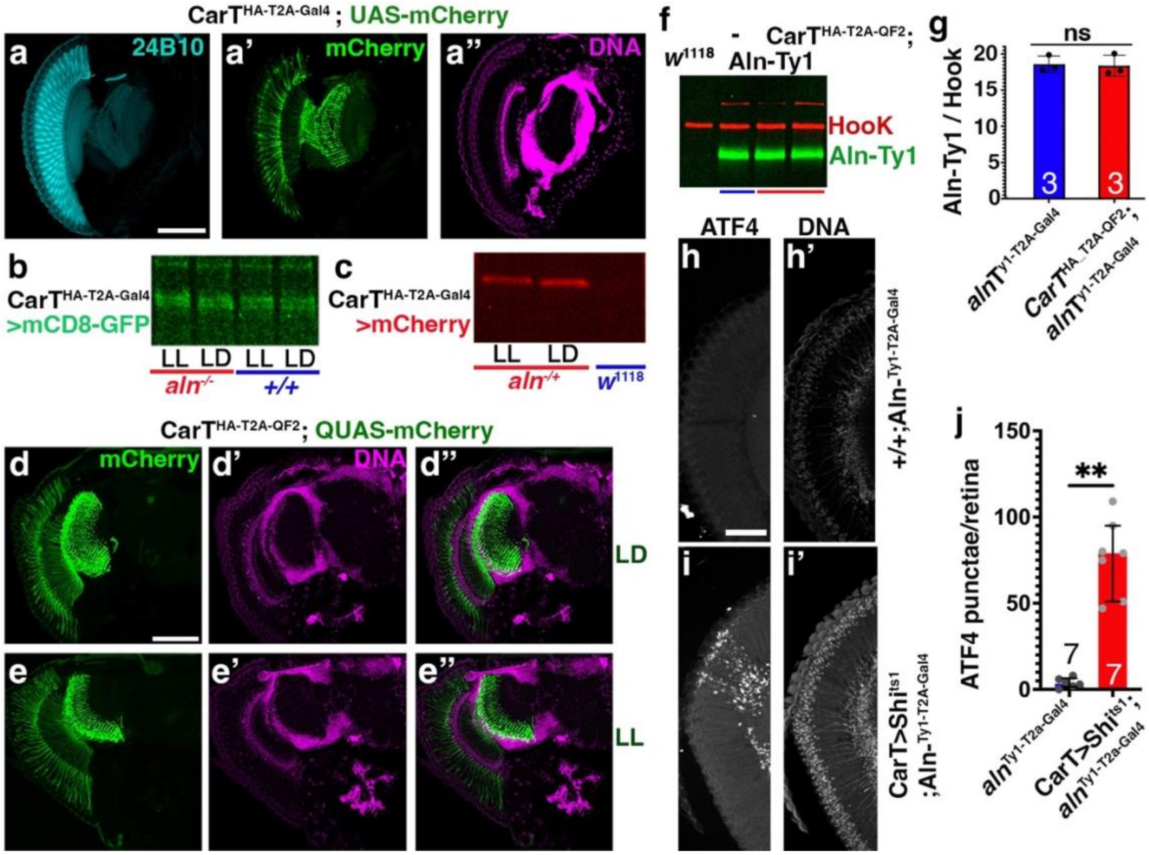

Supplementary Figure 9. The CarT$^{HA\text{-}T2A\text{-}Gal4}$ and CarT$^{HA\text{-}T2A\text{-}QF2}$ drivers are photoreceptor-specific.

Specificity for photoreceptor cells of the two drivers is shown in (a) micrographs of cryosection of adult head stained for DNA, photoreceptor-specific 24B10, and UAS-mCherry expressed under control of CarT$^{HA\text{-}T2A\text{-}Gal}$. (b,c) Western blots of lysates from adult heads expressing UAS-CD8-GFP (b) or UAS-mCherry (c) under CarT$^{HA\text{-}T2A\text{-}Gal}$ control in wild type or *aln* backgrounds treated three days under LD or LL as indicated. (d,e) Micrographs of cryosections of adult heads from flies treated for 3 days under LD (d) or LL (e) stained for DNA and UAS-mCherry expressed under control of CarT$^{HA\text{-}T2A\text{-}QF2}$ driver. Scale bars in a and d are 100 µm and the same for e. Experiments in a-e were repeated twice. The CarT$^{HA\text{-}T2A\text{-}QF2}$ driver does not alter Aln-Ty1 expression as shown in (f) Western blot of lysates from heads from w^{1118}, *aln*$^{Ty1\text{-}T2A\text{-}Gal4}$*, or CarT*$^{Ty1\text{-}T2A\text{-}Gal4}$*; aln*$^{Ty1\text{-}T2A\text{-}Gal4}$ flies probed with antibodies against Ty1 and Hook. (g) Quantification shows no significant CarT$^{HA\text{-}T2A\text{-}QF2}$-dpendent difference of Ty1 expression normalized to the Hook loading control, bar graphs show n and mean ± SD. Statistical significance was assessed using nonparametric Mann-Whitney test, ns, non-significant. QUAS-Shi^{ts1} expression triggers ER stress responses in the retina, as shown by elevated ATF4 expression in micrograph of cryosection of adult heads from aln$^{Ty1\text{-}T2A\text{-}Gal4}$; *CarT*$^{Ty1\text{-}T2A\text{-}QF2}$ flies without (h) or with (i) QUAS-Shi^{ts1} stained for ATF4 or DNA. (j) Quantification shows that Shi^{ts1} expression triggers significant ATF4 accumulation in the retina, graphs show n and median with interquartile range. Bar graph shows n independent samples from two experiments and median with interquartile range. P value determined by nonparametric Mann-Whitney test was 0.0012. Significance threshold for P-Values shown in (g,j) are: ns, non-significant; *,<0.05; **,<0.01.

Supplementary Figure 10

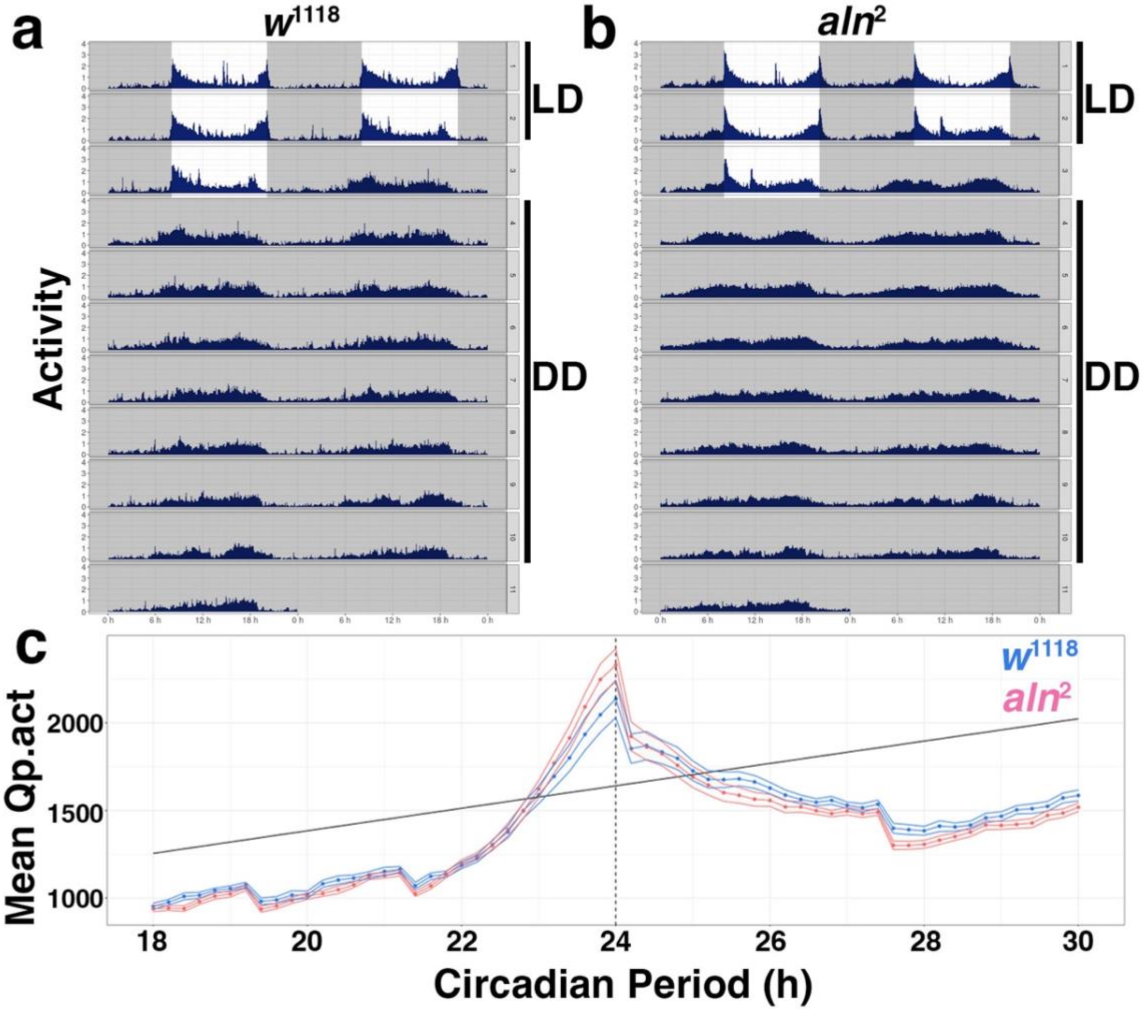

Supplementary Figure 10. Flies lacking *aln* function have normal circadian rhythms under constant darkness (DD).

(a,b) Average activity profiles of (a) w^{1118} or (b) aln^{2} flies (n = 20 flies each) maintained under LD for two days and constant darkness for eight days show normal circadian profiles for aln^{2} flies.

(c) Quantification of amplitude and period by Chi-square periodogram shows both w^{1118} and aln^{2} flies exhibit 24 hr periodicity in DD. The activity was recorded in Drosophila activity monitor (DAM) and analyzed by ShinyR-DAM program [2].

Supplementary Table 1

DNA oligonucleotides used

aln*[1] *mutant gRNAs	
Aln gRNA1 sense	GTCGGGATCCGTGATTCCCGACGA
Aln gRNA1 antisense	AAACTCGTCGGGAATCACGGATCC
Aln gRNA2 sense	GTCGGGACCCTACTACGCCACTGA
Aln gRNA2 antisense	AAACTCAGTGGCGTAGTAGGGTCC
***aln*[1] PCR Confirmation**	
DsRed fwd	ACCTCCCCCTGAACCTGAAA
Aln rev conf	GCGCCTATGTTGAAATGGTT
DsRed rev	AAGGTGTACGTGAAGCACCC
Aln fwd conf	TTTCCTGGTTCTCACGCTCT
***aln*[2] PCR Confirmation**	
Aln fwd conf	TTTCCTGGTTCTCACGCTCT
Aln rev conf	GCGCCTATGTTGAAATGGTT
**Aln-Ty1-T2a-Gal4 tagging gRNAs**	
Aln gRNA3 sense	CTTCGGATCCGAGATTCCCGACGA
Aln gRNA3 antisense	AAACTCGTCGGGAATCTCGGATCC
Aln gRNA4 sense	CTTCGGCGCCTATGTTGAAATGGT
Aln gRNA4 antisense	AAACACCATTTCAACATAGGCGCC
**Aln-Ty1-T2a-Gal4 PCR Confirmation**	
Gal4_fwd	GCGTATAACGCGTTTGGAAT
Aln_rev	AGGTCGAAGGATCGAAGGAT
Gal4_rev	TCGGTTTTTCTTTGGAGCAC
Aln_fwd	AGCAAATGCAAAACGGAAAC
**Aln-Δ-Gal4 gRNAs**	
Aln gRNA3 sense	GTCGGGACCCTACTACGCCACTGA
Aln gRNA3 antisense	AAACTCAGTGGCGTAGTAGGGTCC
Aln gRNA4 sense	GTCGGGATCCGTGATTCCCGACGA
Aln gRNA4 antisense	AAACTCGTCGGGAATCACGGATCC
**Aln-Δ Gal4 PCR Confirmation**	
Gal4_fwd	GCGTATAACGCGTTTGGAAT
Aln_rev	TTGGTAAGCAGGAGCCAGTT
Gal4_rev	TCGGTTTTTCTTTGGAGCAC
Aln_fwd	AAACAGGCACCTCAAAATGG

**genAln rescue**	
genAln_fwd	TCGACGGTCACGGCGGGCATGTCGACTCTAGAGGATCCAGAATGCAACCCGAAAATC
genAln_rev	GTTTTATTAACTTACATACATACTAGAATTCGGTACCAAGGATCGAAGGATCGATG
**CarT-QF2 and CarT-Gal4 gRNAs**	
CarT gRNA1 sense	CTTCGGGTGTCCATGACGCTCATT
CarT gRNA1 antisense	AAACAATGAGCGTCATGGACACCC
CarT gRNA2 sense	CTTCGGTGCCATCATGCGTCCTCT
CarT gRNA2 antisense	AAACAGAGGACGCATGATGGCACC
**CarT-QF2 PCR Confirmation**	
QF2_fwd	CCGTACCACTCGAATCTGGT
CarT_rev	GTTCTCGAGTTTCTGCCCAG
QF2_rev	TCCATCAGCATGAGCGTTAG
CarT_fwd	GCTGGAGGCTCAGATACGAC
**CarT-Gal4 PCR Confirmation**	
Gal4_fwd	GCGTATAACGCGTTTGGAAT
CarT_rev	GTTCTCGAGTTTCTGCCCAG
Gal4_rev	TCGGTTTTTCTTTGGAGCAC
CarT_fwd	GCTGGAGGCTCAGATACGAC
**qPCR Primers**	
RP49_qPCR_Fwd	ATCGGTTACGGATCAAACAA
RP49_qPCR_Rev	GACAATCTCCTTGCGCTTCT
Atg8a_qPCR_Fwd	CGCATCGGTGATTTGGACAA
Atg8a_qPCR_Rev	CTCCTCGTGATGTTCCTGGT

Supplementary Table 2. Genotypes of Flies Used for Each Figure

**Figure 1**	**Genotype**
1 A-J	w^{1118}
1 K-M	w^{*}; ;aln^{1}
1 N-P	w^{1118}<br>w^{*}; ;aln^{1}
**Figure 2**	**Genotype**
2 G	w^{1118},<br>w^{1118}; ; $aln^{\Delta\text{-Gal4}}$,<br>w^{1118}; P[w^{+}, UAS-Aln-Flag]^{43A1}; $aln^{\Delta\text{-Gal4}}$<br>w^{1118}; P[w^{+}, UAS-Dipk1C^{WT}-Flag]^{43A1}; $aln^{\Delta\text{-Gal4}}$<br>w^{1118}; P[w^{+}, UAS-Dipk1C^{D297A}-Flag]^{43A1}; $aln^{\Delta\text{-Gal4}}$<br>w^{1118}; P[w^{+}, UAS-GC11170-Alfa]^{43A1}; $aln^{\Delta\text{-Gal4}}$
**Figure 3**	**Genotype**
3 A-B, E	w^{1118} ; w^{1118} ; p[w^{+}, *tub-Atf4*$^{5'UTR}$-dsRed]/+; +/+
3 C-D, E	w^{1118} ; w^{1118} ; p[w^{+}, *tub-Atf4*$^{5'UTR}$-dsRed]/+; +/+<br>; aln^{2}
3 F	w^{1118} ; p[w^{+}, *tub-Atf4*$^{5'UTR}$-dsRed]/+; +/+ and w^{1118} ; p[w^{+}, *tub-Atf4*$^{5'UTR}$-dsRed]/+; aln^{2}
3 H-I, J-L	w^{1118} and w^{1118} ; ; aln^{2}
3 G	w^{1118} ; *CarT*$^{HA\text{-}T2a\text{-}Gal4}$ / P[w+, UAS^{Scer}-Xbp1-eGFP.hg];+/+ and w^{1118} ; *CarT*$^{HA\text{-}T2a\text{-}Gal4}$ / P[w+, UAS^{Scer}-Xbp1-eGFP.hg]; aln^{2}
3 M-N”, O	w^{1118} and w^{1118} ; ; aln^{2}
**Figure 4**	**Genotype**
5 A-C, F-H	P[GMR_w^{RNAi}]^{5B8}; P[w+,UAS-mCD8-GFP] ,$aln^{Ty1\text{-}T2a\text{-}Gal4}$
5 D-E	w^{1118};+/+; $aln^{Ty1\text{-}T2a\text{-}Gal4}$
5 I	w^{1118}
5 J	w^{1118}; P[w^{+}, UAS-Aln-Flag]^{43A1}; $aln^{\Delta\text{-Gal4}}$
5 K	w^{1118}; P[w^{+}, UAS-DipkC-Flag]^{43A1}; $aln^{\Delta\text{-Gal4}}$
5 L	w^{*}; P[w+, YFP^{MYC}-Rab5]/ +; $aln^{Ty1\text{-}T2a\text{-}Gal4}$ /+
5 M	w^{1118};+/+; $aln^{Ty1\text{-}T2a\text{-}Gal4}$
5 N	w^{*}; P[w+, YFP^{MYC}-Rab11]/+; $aln^{Ty1\text{-}T2a\text{-}Gal4}$ /+
**Figure 5**	**Genotype**
6 A, D, E, F, G, H, M, N, P, Q, R, T	w^{1118};+/+; $aln^{Ty1\text{-}T2a\text{-}Gal4}$
6 B, D, E, F, I, J, M, N	w^{1118} ; *CarT*$^{HA\text{-}T2a\text{-}QF2}$, P[w+, QUAS-Shi^{ts1}]; $aln^{Ty1\text{-}T2a\text{-}Gal4}$
6 C, D, E, O, S, T	w^{1118}
6 K, L, M, N, O	w^{1118} ; *CarT*$^{HA\text{-}T2a\text{-}QF2}$, P[w+, QUAS-TTL]; $aln^{Ty1\text{-}T2a\text{-}Gal4}$ and CarT[43]

Figure 6	Genotype
6 A,D	W*;P{y+w+=5xUAS-DenMark::smGdP-V5}su(Hw)attP5; L4-Gal4
6 B,D	W*;P{y+w+=5xUAS-DenMark::smGdP-V5}su(Hw)attP5; L2-Gal4
6 C ,D,H	W*;P{y+w+=5xUAS-DenMark::smGdP-V5}su(Hw)attP5;
6 E,I	w^{1118} ; $CarT^{HA\text{-}Gal4}$/ P[w^{+}, UAS-Aln-Flag]$^{43A1;}$$aln^{2}$
F,I	w^{1118}
G,I	w^{1118} ; ort-Gal4/ P[w^{+}, UAS-Aln-Flag]$^{43A1;}$$aln^{2}$
**Figure 7**	**Genotype**
7 A-G	w^{1118}<br>w^{1118} ; ; aln^{2}
7 E, F	w^{1118}<br>w^{1118} ; ; aln^{2}<br>w^{1118}; ; $aln^{\Delta\text{-}Gal4}$,<br>w^{1118}; P[w^{+}, UAS-Aln-Flag]^{43A1}; $aln^{\Delta\text{-}Gal4}$

Supplementary Figures	Genotype
**Supplementary Figure** 1	w^{1118} ; ; aln^{2}
**Supplemental Figure 2**	**Genotype**
2B	w^{*} ; ;aln^{1} ,<br>w^{1118}<br>w^{1118} ; ; aln^{2}
2C	w^{1118}<br>w^{1118} ; ; aln^{1}<br>W^{1118}; P[W+, gAln]^{43A1}; aln^{1}
2D	w^{1118}<br>w^{1118} ; ; aln^{1}<br>w^{1118} ; ; $aln^{Ty1\text{-}T2a\text{-}Gal4}$
**Supplementary Figure 4**	**Genotype**
4A,B,E,F, I,J,M,N,O,R,S,V,W, X	w^{1118}
4C,D,K,L,M,P,QT,U, V,W,X	w^{1118} ; ; aln^{2}
4E,F	w^{1118} ; $CarT^{HA\text{-}T2a\text{-}Gal4}$ / P[w+, UAS^{Scer}-Xbp1-eGFP.hg];+/+
4G,H	w^{1118} ; $CarT^{HA\text{-}T2a\text{-}Gal4}$ / P[w+, UAS^{Scer}-Xbp1-eGFP.hg]; aln^{2}
**Supplementary Figure 5**	**Genotype**
5A,C,F,F,G	w^{1118}

5B,D,E,F,G	*w*^{1118} ; ; *aln*^{2}
**Supplementary Figure 6**	**Genotype**
	w^{1118}; *CarT*$^{HA-T2a-Gal4}$ / +; P[v+, uas-Atg1^{RNAi}]/ +<br>*w*^{1118}; *CarT*$^{HA-T2a-Gal4}$ / +; P[v+, uas-Atg5 ^{RNAi}]/ +<br>*w*^{1118}; *CarT*$^{HA-T2a-Gal4}$ / +; P[v+, uas-Atg9 ^{RNAi}]/ +<br>*w*^{1118}; *CarT*$^{HA-T2a-Gal4}$ / +; P[v+, uas-At18 ^{RNAi}]/ +<br>*w*^{1118}
**Supplementary Figure 7**	**Genotype**
7A,C, D and E	*w*^{1118}
7B,C, D and F	*per*^{01}
**Supplementary Figure 8**	**Genotype**
A-B	*w*^{1118} ; P[y+ w+; 20xUAS-6xmCherry-HA]^{VK18}/ P[y+,w+, 10xQUAS-6xGFP]^{VK18}; p[w+, nSyb-QF2.P]^{attP2} / *aln*$^{Ty1-T2a-Gal4}$
C	*w*^{1118}; ; *aln*$^{\Delta-Gal4}$/UAS-NLS::mCherry
**Supplemental Figure S9**	**Genotype**
9A	*w**; *CarT*$^{HA-T2a-Gal4}$/+; P[y+ w+; 20xUAS-6XmCherry]^{attP2}/+
9B	*w**; *CarT*$^{HA-T2a-Gal4}$/+; P[y+ w+; 10xUAS-mCD8::GFP]
9C	*w**; *CarT*$^{HA-T2a-Gal4}$/+; P[y+ w+; 20xUAS-6XmCherry]^{attP2}/+<br>*w*^{1118}
9D,E	*w**; *CarT*$^{HA-T2a-QF2}$/+; QUAS-mCherry
9F,G	*w*^{1118} ; ; *aln*$^{Ty1-T2a-Gal4}$<br>*w*^{1118} ; *CarT*$^{HA-T2a-QF2}$; *aln*$^{Ty1-T2a-Gal4}$
9H,J	*w*^{1118} ; ; *aln*$^{Ty1-T2a-Gal4}$
9I, J	*w*^{1118} ; *CarT*$^{HA-T2a-QF2}$, P[w+, QUAS-Shi^{ts1}]; *aln*$^{Ty1-T2a-Gal4}$
**Supplemental Figure S10**	**Genotype**
10	*w*^{1118}<br>*w*^{1118} ; ;*aln*^{2}

Supplementary references:

1 Crooks, G. E., Hon, G., Chandonia, J. M. & Brenner, S. E. WebLogo: a sequence logo generator. *Genome Res* **14**, 1188-1190, doi:10.1101/gr.849004 (2004).

2 Cichewicz, K. & Hirsh, J. ShinyR-DAM: a program analyzing Drosophila activity, sleep and circadian rhythms. *Commun Biol* **1**, 25, doi:10.1038/s42003-018-0031-9 (2018).

Supplementary Figure 4W full blot

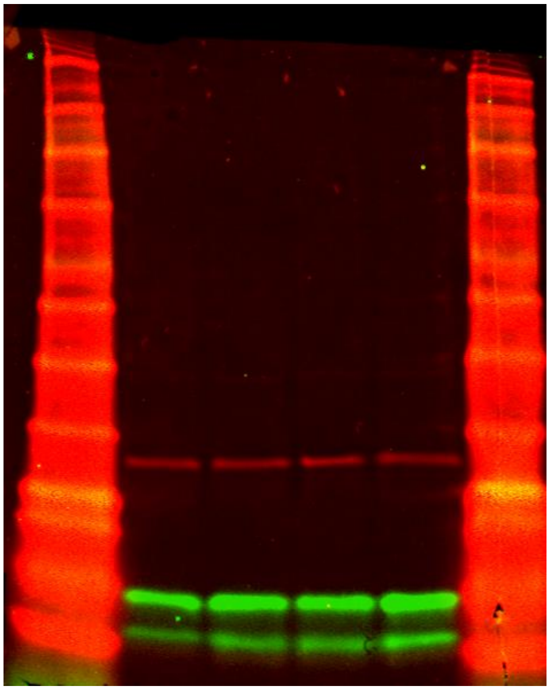

Supplementary Figure 5 F full blot

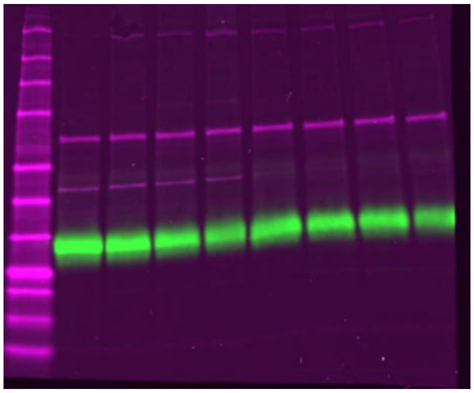

Supplementary Figure 6 full blot

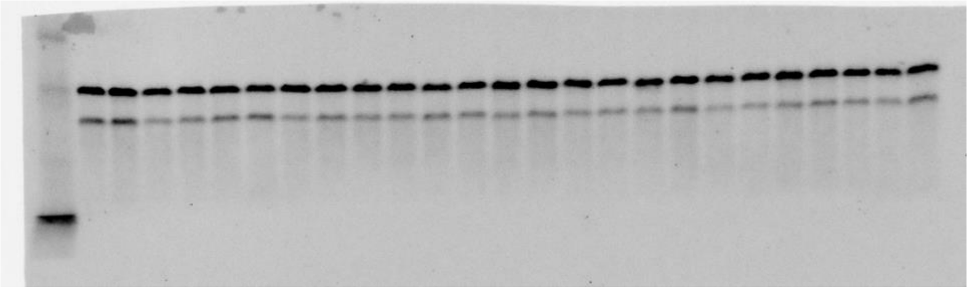

Supplementary Figure 9 full blots

9B

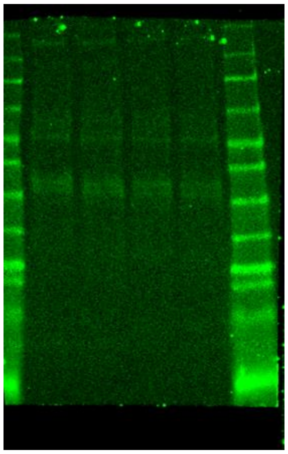

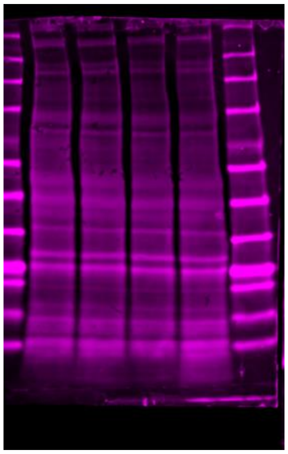

9C

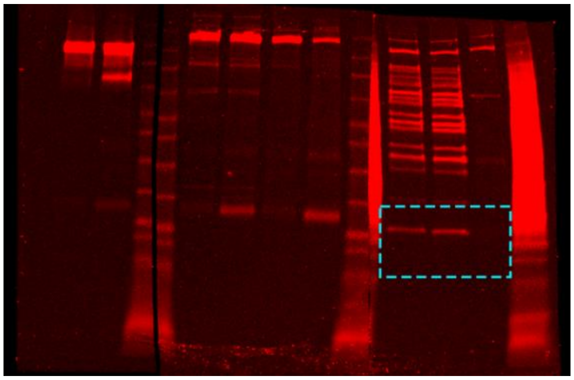

9f

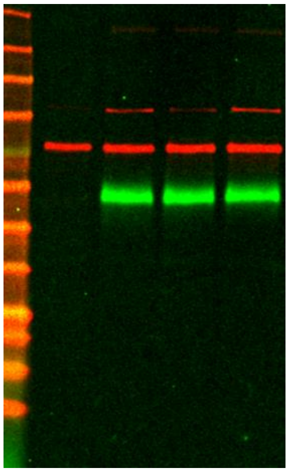

Supplement: Supplementary file 1 — Supplementary Information [file 41467_2023_38485_MOESM1_ESM.pdf]
