# Supplementary material for: Allnighter pseudokinase-mediated feedback links proteostasis and sleep in Drosophila
Source: Nat Commun. 2023 May 22;14:2932. doi: 10.1038/s41467-023-38485-7 (PMC10203134; doi:10.1038/s41467-023-38485-7)
Supplement: Supplementary file 3 — Reporting Summary [file 41467_2023_38485_MOESM3_ESM.pdf]

nature portfolio

Corresponding author(s): Helmut Krämer, Shashank Shekhar

Last updated by author(s): April 26, 2023

Reporting Summary

Nature Portfolio wishes to improve the reproducibility of the work that we publish. This form provides structure for consistency and transparency in reporting. For further information on Nature Portfolio policies, see our Editorial Policies and the Editorial Policy Checklist.

Statistics

For all statistical analyses, confirm that the following items are present in the figure legend, table legend, main text, or Methods section.

n/a	Confirmed	
☐	☒	The exact sample size (*n*) for each experimental group/condition, given as a discrete number and unit of measurement
☐	☒	A statement on whether measurements were taken from distinct samples or whether the same sample was measured repeatedly
☐	☒	The statistical test(s) used AND whether they are one- or two-sided<br>*Only common tests should be described solely by name; describe more complex techniques in the Methods section.*
☐	☒	A description of all covariates tested
☐	☒	A description of any assumptions or corrections, such as tests of normality and adjustment for multiple comparisons
☐	☒	A full description of the statistical parameters including central tendency (e.g. means) or other basic estimates (e.g. regression coefficient) AND variation (e.g. standard deviation) or associated estimates of uncertainty (e.g. confidence intervals)
☐	☒	For null hypothesis testing, the test statistic (e.g. *F*, *t*, *r*) with confidence intervals, effect sizes, degrees of freedom and *P* value noted<br>*Give P values as exact values whenever suitable.*
☒	☐	For Bayesian analysis, information on the choice of priors and Markov chain Monte Carlo settings
☒	☐	For hierarchical and complex designs, identification of the appropriate level for tests and full reporting of outcomes
☒	☐	Estimates of effect sizes (e.g. Cohen's *d*, Pearson's *r*), indicating how they were calculated

Our web collection on statistics for biologists contains articles on many of the points above.

Software and code

Policy information about availability of computer code

Data collection	Ethoscope arena (Geissmann et al., 2017) was used to record the sleep behavior which is an open platform created by Gilestro lab.
Data analysis	EM images were procured using a JEOL 1400 Plus and quantified in ImageJ (NIH), The traces of ERG were collected by an electrometer (IE-210; Warner Instruments, Hamden, CT), digitized with a Digidata 1440A and MiniDigi 1B system (Molecular Devices, San Jose, CA), and recorded using Clampex 10.2 (Molecular Devices) and quantified with Clampfit software (Molecular Devices). Confocal mages were captured with a 20x NA-0.8 or an oil-immersion 63× NA-1.4 lens on an inverted confocal microscope (LSM710 or LSM880 with Airyscan, Carl Zeiss) and quantified in ImageJ V. 2.1.0 /1.53c (NIH) or Imaris 9.5 (Oxford Instruments). Western blot images were taken in Odyssey scanner (LI-COR Biosciences) and quantified in EmpiriaStudio Version 2.3.0.154 (2.3.0.154). Sleep data was analyzed using rethomics an R-based open source platform. All the statistical analysis were done by using Prism 9 Version 9.1.2 (225), GraphPad Software, LLC. Protein structure visualization: PyMol V. 2.5.5. For protein sequence alignments: Mafft V.7.463. We added this info:<br>The modified code to load, clean, and analyze the sleep data was initially adapted from https://rethomics.github.io/ 33,34. Additional code was written to measure Zt-12 latency to sleep. All codes are deposited at github https://github.com/mz27ethio/Ethoscope-Kramerlab.git.

For manuscripts utilizing custom algorithms or software that are central to the research but not yet described in published literature, software must be made available to editors and reviewers. We strongly encourage code deposition in a community repository (e.g. GitHub). See the Nature Portfolio guidelines for submitting code & software for further information.

Data

Policy information about availability of data

All manuscripts must include a data availability statement. This statement should provide the following information, where applicable:

- Accession codes, unique identifiers, or web links for publicly available datasets
- A description of any restrictions on data availability
- For clinical datasets or third party data, please ensure that the statement adheres to our policy

Sleep data are available in the Zenodo database under accession code (DOI: 10.5281/zenodo.7843827). All other data generated or analysed during this study are included in this published article (and its supplementary information files). Source data are provided with this paper.
Databases used in this study NCBI refseq protein (https://www.ncbi.nlm.nih.gov/refseq/); Flybase (http://flybase.org); Bloomington Drosophila stock center (https://bdsc.indiana.edu).

Human research participants

Policy information about studies involving human research participants and Sex and Gender in Research.

Reporting on sex and gender	not applicable
Population characteristics	not applicable
Recruitment	not applicable
Ethics oversight	not applicable

Note that full information on the approval of the study protocol must also be provided in the manuscript.

Field-specific reporting

Please select the one below that is the best fit for your research. If you are not sure, read the appropriate sections before making your selection.

☒ Life sciences ☐ Behavioural & social sciences ☐ Ecological, evolutionary & environmental sciences

For a reference copy of the document with all sections, see nature.com/documents/nr-reporting-summary-flat.pdf

Life sciences study design

All studies must disclose on these points even when the disclosure is negative.

Sample size	No sample size was predetermined. Samples sizes were chosen according to the standards generally accepted in the field
Data exclusions	No data were excluded from analysis
Replication	All replication attempts were successful and each experiment was repeated at least two times.
Randomization	For each experimental condition in each genotype flies were collected on the same day and same time but before treatment to experimental conditions, randomly distributed in different vials according to genotype and then labeled for the treatment conditions without any specific preferences.
Blinding	Blinding of samples before quantification was done wherever it was possible. For the sleep and ERG experiments experimenter was blinded to genotype during group allocation. For antibody staining experiments, experimenter was blinded during data collection. Any experiments done without blinding (especially during the Covid19 epidemic) were repeated at least twice with blinding.

Reporting for specific materials, systems and methods

We require information from authors about some types of materials, experimental systems and methods used in many studies. Here, indicate whether each material, system or method listed is relevant to your study. If you are not sure if a list item applies to your research, read the appropriate section before selecting a response.

Materials & experimental systems

n/a	Involved in the study
	☒ Antibodies
	☒ Eukaryotic cell lines
☒	Palaeontology and archaeology
	☒ Animals and other organisms
☒	Clinical data
☒	Dual use research of concern

Methods

n/a	Involved in the study
☒	ChIP-seq
☒	Flow cytometry
☒	MRI-based neuroimaging

Antibodies

Antibodies used

rabbit Anti-GABARAP (Abcam [EPR4805] (ab109364), Mouse anti-Ty1 clone BB2 (Thermo Fisher Scientific; MA5-23513), rabbit anti-Hook (krämer lab made), mouse anti-Actin (JLA20, DSHB:JLA20; RRID: AB_528068), rabbit anti-RFP (Rockland:600-401-379; RRID:AB_2209751), Mouse anti-GFP clone B2 (Rockland:600-401-379; RRID:AB_2209751), anti-GFP (Chicken polyclonal, ThermoFisher Scientific:A10262; RRID: AB_2534023) anti-Hsc70-3 (BiP) gift from Don Ryoo, NYU (Guinea Pig polyclonal, PMID:17170705), M2 anti-Flag (mouse monoclonal, Sigma:F-3165; RRID:AB_259529), Anti-RH1 (mouse monoclonal DSHB, 4C5-c), Anti-24b10 (rabbit polyclonal), Anti-TRP (mouse monoclonal, DSHB,MAb83F6), Anti-DIP-Beta (Guinea Pig polyclonal, Gift from Dr. Matthew Pecot, Harvard Medical school, PMID: 31300277), Anti-ATF4 (rat polyclonal,Gift from Dr. Joseph Bateman, King's College, PMID: 31645461), Anti-ELAV(mouse monoclonal ,DSHB, 9F8A9-c) . Alexa 488- or 568- or 647 secondaries (Molecular Probes), STAR-Red Secondaries (Abberior), LICOR 800 or 700- secondaries (LICOR Biosciences)

Validation

Validation
anti-Hsc70-3 (BiP) (Guinea Pig polyclonal) ref. 63 FLYB: FBgn0001218; RRID: AB_2569409 Gift from Don Ryoo, NYU (1:1000 IHC)
Reference paper validated for IHC and western blot and confirmed by us in western blots.

anti-RFP (Rabbit polyclonal) Rockland Rockland:600-401-379; RRID:AB_2209751
(1:500 IHC, WB 1:500) Manufacturer validated. Suitable for: WB, IHC. Specificity confirmed in our lab with negative controls in WB and IHC
anti-GFP (Chicken polyclonal). ThermoFisher Scientific ThermoFisher Scientific:A10262; RRID: AB_2534023
(1:1000 IHC) Manufacturer validated. Suitable for: IHC. Specificity confirmed in our lab with negative controls in WB and IHC

anti-GFP (Mouse monoclonal, B2) Santa Cruz Santa Cruz Anti-GFP Antibody (B-2): sc-9996 (1:50 IHC, 1:250 WB) Manufacturer validated. Suitable for: WB, IHC. Specificity confirmed in our lab with negative controls in WB and IHC.

M2 anti-Flag (mouse monoclonal, clone M2) Sigma Sigma: F-3165; RRID:AB_259529. (1:1000 IHC, 1:2000 WB) Specificity confirmed in our lab with negative controls in WB and IHC

Anti-GABARAP Abcam Abcam [EPR4805] (ab109364) (1:200 IHC, 1:1000 WB) Manufacturer validated. Suitable for: Flow Cyt (Intra), WB, IHC-P, ICC/IF. We validated in IHC of Drosophila tissue by showing increased in staining by CQ treatment and other methods known to enhance autophagy and by correct size by western blot.

anti-Actin (mouse monoclonal) Developmental Studies Hybridoma Bank DSHB:JLA20; RRID: AB_528068
1:2000 (WB) JLA20 was deposited to the DSHB by Lin and validated for western blot as per DSHB references.

Anti-HA (Mouse monoclonal) MBL mbl TANA2 HA antibody (1:1000 IHC, 1:3000 WB) Specificity confirmed in our lab with negative controls in WB and IHC

Alexa 488- or 568- or 647 secondaries
Alexa Fluor 568 Goat anti-mouse
Alexa Fluor 488 Goat anti-mouse
Alexa Fluor 647 Goat anti-mouse
Alexa Fluor 488Goat anti-rabbit
Alexa Fluor 568 Goat anti-rabbit
Alexa Fluor 647 Goat anti-rabbit
Alexa Fluor 647 Goat anti-guinea pig
Alexa Fluor 568 Goat anti-guinea pig
Alexa Fluor 488 Goat anti-guinea pig
Alexa Fluor 668 Goat anti-rat
Alexa Fluor 488 Goat anti-chicken
ThermoFisher thermofisher fluorescent-secondary-antibodies
(1:500 IHC) Manufacturer validated for cell Imaging and confirmed in experiments with negative controls in IHC.

Goat anti-Mouse STAR-Red Secondaries Abberior https://www.fishersci.com/shop/products/star-red-goat-anti-mouse-igg/NC1933868. (1:500 IHC) Manufacturer validated and confirmed in experiments with negative controls in IHC.

IRDye 800CW Goat anti mouse
IRDye 700DX Goat anti mouse
IRDye 800CW Goat anti Rabbit
IRDye 700DX Goat anti Rabbit

LICOR Biosciences https://www.licor.com/bio/reagents/new-reagent=category=irdye-secondary-antibodies
(1:20,000 WB) Manufacturer validated. and confirmed in our lab with negative controls in western blots

Anti-Ty1 (mouse monoclonal, BB2) Invitrogen Thermo Fisher Scientific; MA5-23513
(1:500 IHC, 1:2000 WB) Specificity confirmed in our lab with negative controls in WB and IHC

Anti-RH1 (mouse monoclonal DSHB) Developmental Studies Hybridoma Bank https://dshb.biology.uiowa.edu/4C5
(1:500 IHC, 1:1000 WB) 4C5 was deposited to the DSHB by de Couet, H.G. / Tanimura, T. and validated for western blot as per DSHB references and in our lab with negative controls.
Anti-24b10 (rabbit polyclonal) Developmental Studies Hybridoma Bank https://dshb.biology.uiowa.edu/24B10
1:2000 24B10 was deposited to the DSHB by Benzer, S. and validated for known IHC pattern.

Anti-TRP (mouse monoclonal) Developmental Studies Hybridoma Bank https://dshb.biology.uiowa.edu/MAb83F6
1:300 IHC MAb83F6 was deposited to the DSHB by Benzer, S. and validated for IHC and western blot as per DSHB references.

Anti-DIP-Beta (Guinea Pig polyclonal) ref. 29 Gift from Dr. Matthew Pecot, Harvard Medical school 1:250 IHC Reference paper validated for IHC and confirmed by us for identical staining pattern.

Anti-ATF4 (rat polyclonal) ref. 29,64 Gift from Dr. Joseph Bateman, King's College 12000 IHC Reference paper validated for IHC and confirmed by us for identical staining pattern.

Anti-ELAV (mouse monoclonal) Developmental Studies Hybridoma Bank https://dshb.biology.uiowa.edu/Elav-9F8A9
1:10000 IHC Elav-9F8A9 was deposited to the DSHB by Rubin, Gerald M and validated for IHC and confirmed by us for identical staining pattern.

Anti-Hook (rabbit polyclonal) lab generated 1:5000 WB, 1:1000 IHC Antibody was validated by using hook null mutants as negative control in WB.

Eukaryotic cell lines

Policy information about cell lines and Sex and Gender in Research

Cell line source(s)	HEK293 cells were a generous gift of the Dixon lab (UCSD), not commercially sourced
Authentication	In this study HEK293 cells were exclusively used for the production and subsequent purification of proteins. No further authentication was used.
Mycoplasma contamination	the cells are routinely tested for mycoplasma using a sensitive PCR-based assay. Cells used in this study tested negative for mycoplasma.
Commonly misidentified lines (See ICLAC register)	No commonly misidentified cell lines were used.

Animals and other research organisms

Policy information about studies involving animals; ARRIVE guidelines recommended for reporting animal research, and Sex and Gender in Research

Laboratory animals	Animals used in this study are listed in table in method section in details. many wild type and transgenic strains were obtained from Bloomington Stock Center and peers in the Drosophila scientific community. Some flies were generated in this study. All the details about origin and species are detailed in table in method section.<br>Unless otherwise stated flies were 3 to 5 days old when used for experiments
Wild animals	This study did not involve wild animals.
Reporting on sex	Males and females both are included in this study depending on the experimental condition. For example EEG recordings are only done in females whereas sleep experiments were done in males. Details about sex is specifically mentioned in method section.
Field-collected samples	No field collected animals were used in this study.
Ethics oversight	No ethical approval was required because we used Drosophila melanogaster which is not regulated by The Animal (Scientific procedure) Act 1986.

Note that full information on the approval of the study protocol must also be provided in the manuscript.
